# Structural basis for the bacterial membrane insertion of dermcidin peptide, DCD-1L

**DOI:** 10.1038/s41598-017-13600-z

**Published:** 2017-10-24

**Authors:** Van Sang Nguyen, Kang Wei Tan, Karthik Ramesh, Fook Tim Chew, Yu Keung Mok

**Affiliations:** 1 Department of Biological Sciences, 14 Science Drive 4, National University of Singapore, 117543 Singapore, Singapore; 2 Department of Genetics, Faculty of Biological Sciences, Vietnam National University in Hanoi, 334 Nguyen Trai St., Thanh Xuan, Hanoi Vietnam

## Abstract

Human dermcidin (DCD) is an antimicrobial peptide secreted constitutively by sweat glands. The anionic derivative, DCD-1L, comprises of the N-terminal 47 residues of DCD and one additional leucine residue. A previous NMR structure of DCD-1L in 50% TFE showed a partial helical conformation, and its crystal structure in the presence of Zn^2+^ outlined a hexameric linear α-helical bundle. Three different models to describe membrane insertion were proposed but no conclusion was drawn. In the current study, the NMR structure of DCD-1L in SDS micelles showed an “L-shaped” molecule with three fully formed α-helices connected by flexible turns. Formation of these helices in DCD-1L in the presence of POPG vesicles suggests that the acidic C-terminal region of DCD-1L can suppress the binding of DCD-1L to POPG vesicles at basic but not acidic pH. Mutation of charged residues on the N-terminal and C-terminal regions of DCD-1L cause differences in POPG binding, suggesting distinct functional roles for these two regions. Charged residues from these two regions are also found to differentially affect Zn^2+^ coordination and aggregation of DCD-1L in the absence or presence of SDS, as monitored by 1D NMR. Our data agrees with one of the three models proposed.

## Introduction

Human dermcidin (DCD) is an antimicrobial peptide secreted constitutively by eccrine sweat glands^1^. DCD (110 residues) is first proteolytically processed to generate DCD-1L (N-terminal 48 residues of DCD)^2,3^. Subsequently, DCD-1L is C-terminal processed by cathepsin D, in combination with a 1,10-phenanthroline-sensitive carboxypeptidase and an endoprotease, to yield DCD-1L derived peptides, including SSL-25, and SSL-29 (DCD-1L lacking the C-terminal 23 and 19 residues, respectively)^4^. DCD-1L is anionic, with a net charge of −5, and is amphipathic in nature, whereas SSL-25 is cationic and SSL-29 carries no net charge^1,5^. DCD-1L is predicted to have three helical regions and its cationic N-terminal part is proposed to interact with negatively charged phospholipid membranes of bacteria^5,6^. DCD-1L is monomeric in solution, but can be induced to form oligomers in the presence of Zn^2+^ or ion channels in the presence of bacterial membranes^5^. Because it is an anionic antimicrobial peptide, DCD-1L behaves differently to cationic antimicrobial peptides and has antimicrobial activity over a broad pH range and at high salt concentrations^1,6^. DCD-1L has microbicidal activity against both Gram-positive (*Staphylococcus aureus*) and Gram-negative (*Escherichia coli*) bacteria but shows no hemolytic activity against red blood cells^7^. DCD forms part of the innate defense system^8^ and the reduced levels of DCD peptides or the protease inhibitor, lymphoepithelial Kazal-type inhibitor (LEKTI), in sweat is usually associated with development of diseases like atopic dermatitis^9,10^ and tinea pedis^11^. Patients with atopic dermatitis patients suffer from recurrent skin infections and pronounced colonization of *S*. *aureus* ^9^.

Both solution NMR and crystal structures for DCD-1L have been determined. The solution NMR structure was solved in 50% trifluoroethanol (TFE) without Zn^2+^, revealing 4 highly flexible amphipathic α-helices (E5–G7, G10–K12, E27–S31 and V37–V43) that formed a helix-hinge-helix motif. The N-terminal half (S2–K23) of DCD-1L is more unstructured as compared with the C-terminal portion (D24–L48). Furthermore, the membrane affinity is found to depend on the amphipathic structure, helical content and length of the DCD peptide, rather than its negative net charge or isoelectric point^12^.

The crystal structure of DCD-1L was also determined in the absence of detergent, lipid micelles or membranes but with Zn^2+^ and showed a hexameric α-helical bundle formed by trimerization of anti-parallel peptide dimers^13^. The residue H38 and other acidic residues were proposed to coordinate Zn^2+^, which is essential to induce oligomerization and stabilize the secondary interface.

On the bacterial membrane, studies suggest that the DCD-1L peptide first aligns flat with surface and then slowly forms oligomeric complexes, which are stabilized by Zn^2+^ and coordinated by the H38 residue. The oligomeric complex then breaks open, inserts into the membrane, and undergoes re-oligomerization to form the channel^6^.

Currently, there are three models for DCD-1L membrane insertion: (1) The positively charged N-terminus is embedded in the lipid bilayer while the negatively charged C-terminus floats on the membrane surface^14,15^; (2) The cationic N-terminus of DCD-L is folded back toward the anionic C-terminal region to form a transmembrane hairpin, based on an “electrostatic charge-zipper” mechanism^16^; and (3) The DCD-1L is straight and tilted, maintaining the channel entirely within the membrane^13^. Indeed, through the use of simulations, the authors suggested that ions can enter sideways into the pore through eyelets at the trimeric interface as a result of this channel tilt^13^.

In the current study, we determined solution NMR structures of DCD-1L in 60 mM sodium dodecyl sulfate (SDS) micelles without Zn^2+^, and found a much higher helical content than that found by Jung and colleagues in 50% TFE^12^. The structure of DCD-1L in the SDS micelle showed an “L-shaped” molecule with three α-helices connected by flexible turns. The N-terminal domain is amphipathic and cationic, whereas the C-terminal domain is acidic and contains positively and negatively charged residues on opposite surfaces. With increasing concentrations of phosphatidylglycerol, POPG, which mimics the negatively charged bacterial membrane, we observed a helical conformation for DCD-1L using FarUV-CD. Further, single-site mutants of DCD-1L revealed that the acidic C-terminal domain of DCD-1L reduces the affinity of DCD-1L to POPG, but this could be restored by making the pH more acidic. Mutating charged residues on the N- and C-terminal domains caused drastic differences in POPG binding; these charged residues are unlikely to be involved in simply forming salt-bridges between the two domains within or between molecules. We further studied Zn^2+^ coordination and aggregation of DCD-1L in the absence or presence of SDS using 1D NMR. Overall, our findings show that residues from distinct regions of DCD-1L differentially affect the aggregation of the protein. Based on our findings, we support a model proposed for DCD-1L membrane insertion.

## Results

### NMR solution structure of DCD-1L in SDS

**Figure 1:**
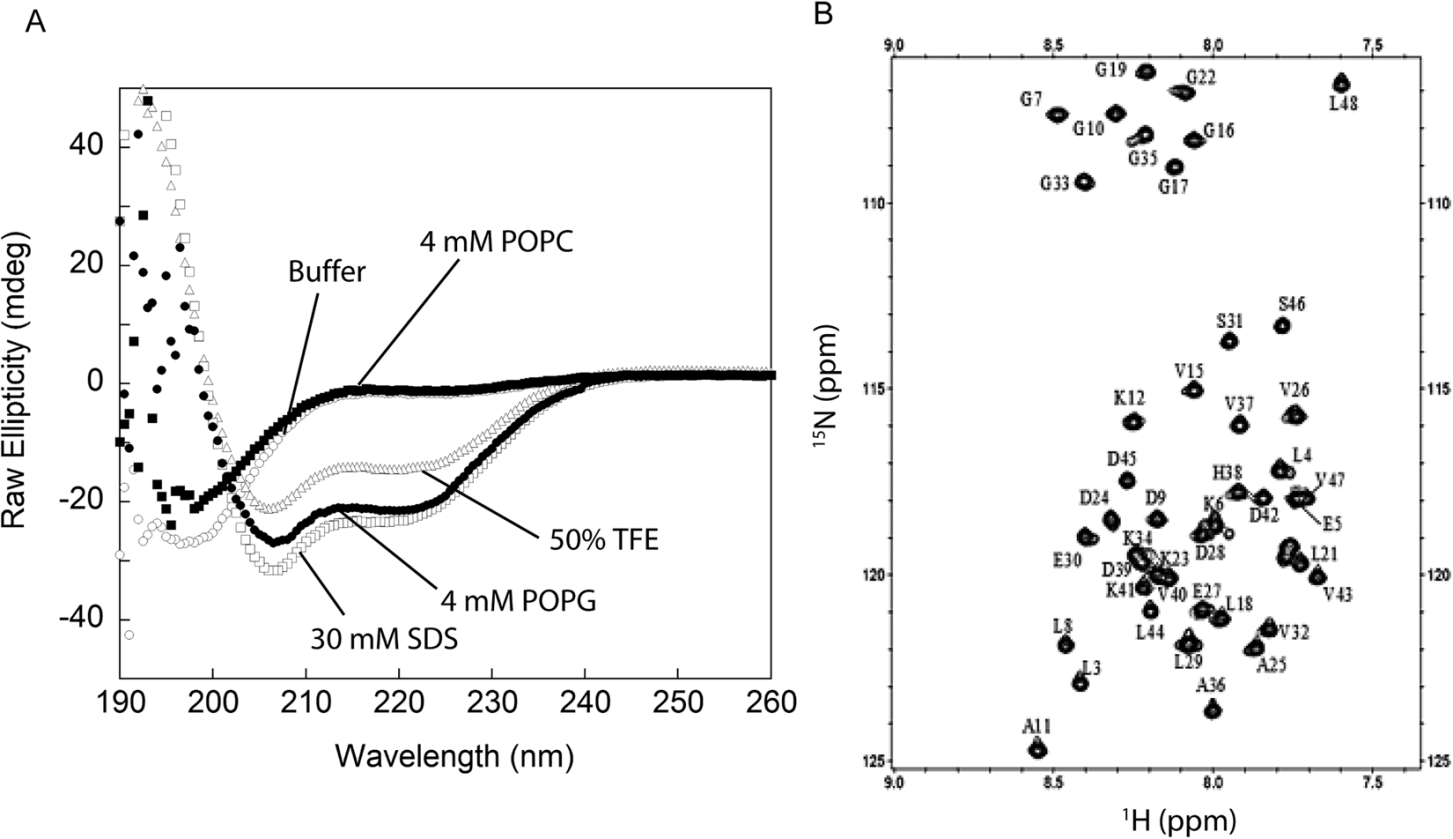
FarUV-CD and NMR ^1^H-^15^N HSQC spectra of DCD-1L. (**A**) FarUV-CD spectra of 30 μM DCD-1L in 20 mM Tris-HCl, pH 7.5 and 0.1 M NaCl (open circle); 4 mM POPC (closed square); 50% TFE (open triangle); 4 mM POPG (closed circle); and 30 mM SDS (open square). (**B**) ^1^H-^15^N HSQC spectrum of DCD-1L in 60 mM deuterated SDS without Zn^2+^ acquired using a 800-MHz NMR at 25 °C. The label next to each amide cross-peak shows the sequence specific assignment.

Far-UV CD spectra of DCD-1L in 20 mM Tris-HCl buffer at pH 7.4 showed a predominantly random coiled conformation (single minimum at 198 nm) (Fig. 1A). In the presence of 4 mM POPG vesicles, which mimic the negatively charged bacterial membrane, DCD-1L adopted an α-helical conformation (double minima at 208 and 222 nm). Interestingly, such conformational change is not observed in the presence of 4 mM phosphatidylcholine (POPC) vesicles, which mimic the neutrally charged eukaryotic cell membrane (Fig. 1A). A previously NMR structure of DCD-1L in 50% TFE showed that DCD-1L contains four highly flexible amphipathic α-helices that form a helix-hinge-helix motif, and that the N-terminal half was much more unstructured than the C-terminal half^12^. Comparing the two structures, we suggest that perhaps the full extent of the helical conformation cannot be achieved with 50% TFE as compared to with that in 4 mM POPG. Indeed, in the presence of 30 mM SDS micelles, which are also negatively charged, and α-helical conformation is adopted similar to that observed with 4 mM POPG (Fig. 1A). These findings prompted us to determine the NMR structure of DCD-1L in the presence of SDS.

**Figure 2:**
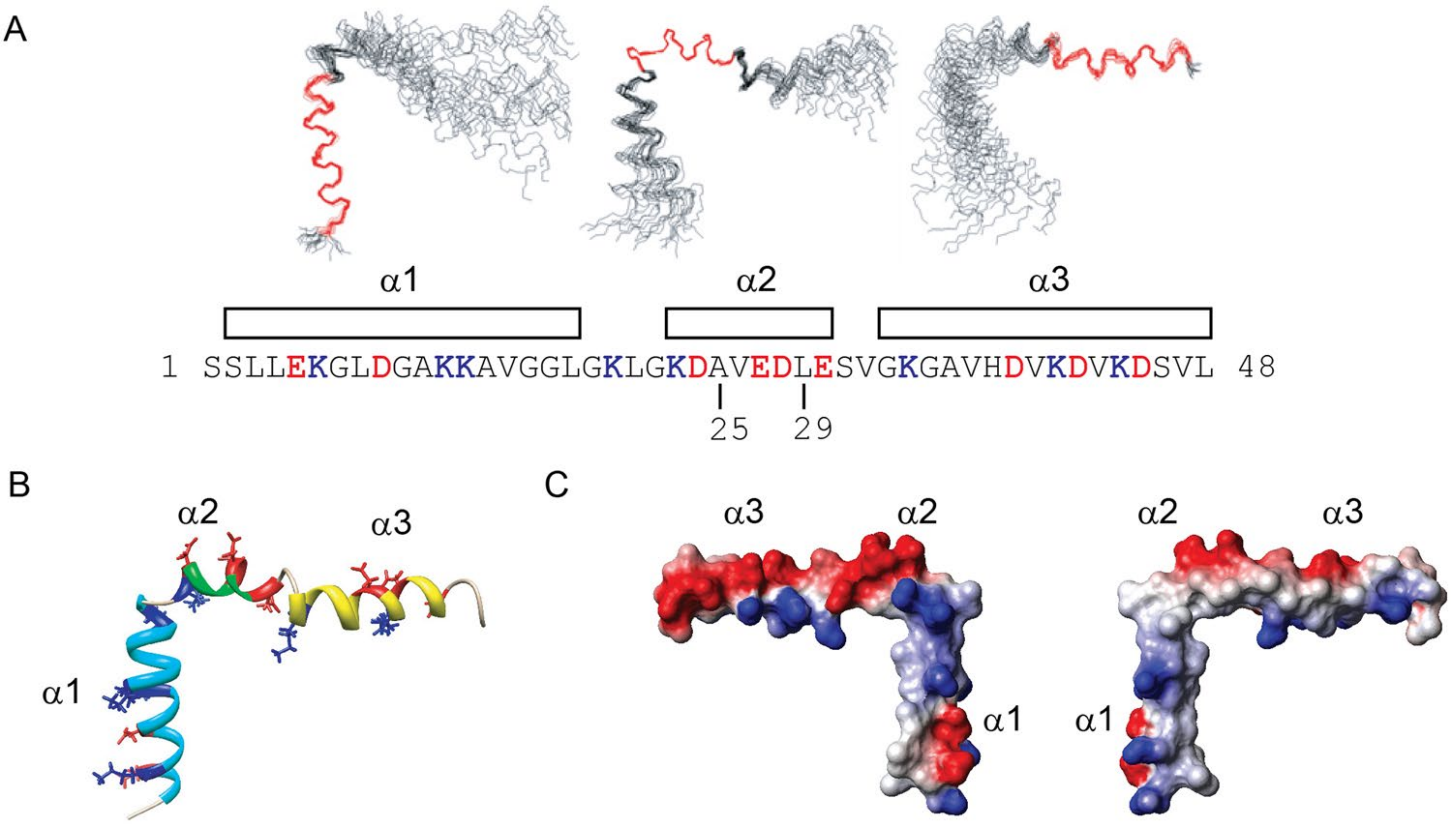
NMR solution structure of DCD-1L in 60 mM SDS without Zn^2+^. (**A**) Backbone diagram of ensembles of 20 NMR structures of DCD-1L superimposed and aligned using α1 (Ser2 to Leu18), α2 (Lys23 to Glu30), or α3 (Gly33 to Leu47). The boundaries of the secondary structures are shown above the sequence of DCD-1L. Positively charged residues are blue and negatively charged residues are red. The C-termini of SSL-25 and SSL-29 are marked on the sequence. (**B**) Ribbon diagram of the NMR structure of DCD-1L showing the three α-helices (α1: cyan; α2: green; α3: yellow). The charged residues are shown as framework model and colored differently (positively charged: blue; negatively charged: red). (**C**) Surface diagrams of DCD-1L at two different views showing different distribution of surface charges (positive: blue; negative: red) at the N- and C-terminal regions of the molecule.

In the presence of SDS, the cross-peaks in the ^1^H-^15^N HSQC spectrum of DCD-1L are well spread out, with similar peak widths and intensities for most residues (45 of 48 peaks can be observed) (Fig. 1B). Unfortunately, we could not determine the NMR structure of DCD-1L in POPG as most of the peaks were missing in the ^1^H-^15^N HSQC spectrum (data not shown). Using various NMR triple resonance and NOESY experiments, the ^1^H-^15^N HSQC spectrum of DCD-1L in SDS was assigned (94% of all amide cross-peaks, only residues S1, S2 and V14 were missing and not assigned) and an ensemble of the structure of DCD-1L was calculated. The detailed NMR structural parameters/statistics (see Supplementary Table S1) and the NOE pattern of the DCD-1L structure (see Supplementary Fig. S1) can be found in the supplemental materials. The overall structure of DCD-1L in SDS is “L-shaped”, consisting of three well-formed α-helices (α1 from residues S2 to L18; α2 from residues K23 to E30; and α3 from residues G33 to L47) connected by two flexible turns (turn 1 from residues L21 to K23 and turn 2 from residues E30 to G33) (Fig. 2A). The flexible turns between the α-helices are Gly-rich or adjacent to Gly-rich region, e.g. ^16^GGLGKLGK ^23^ and ^30^ ESVGKG^35^. The structure is significantly different from that in 50% TFE. Essentially, the mostly unstructured N-terminal domain in 50% TFE is well structured in SDS and forms a single α-helix instead of two short helices.

The N-terminal region of the L-shaped DCD-1L molecule contains one helix (α1), whereas the C-terminal region consists of two helices (α2 and α3) (Fig. 2B). The surface charge plot shows that the N-terminal region is overall cationic and amphipathic, with charged and hydrophobic residues on opposite sides. In contrast, the C-terminal region is anionic overall, and contains positively charged residues on one side and negatively charged residues on the other (Fig. 2B and C).

### Affinity of DCD peptides to POPG as monitored by α-helical content

**Figure 3:**
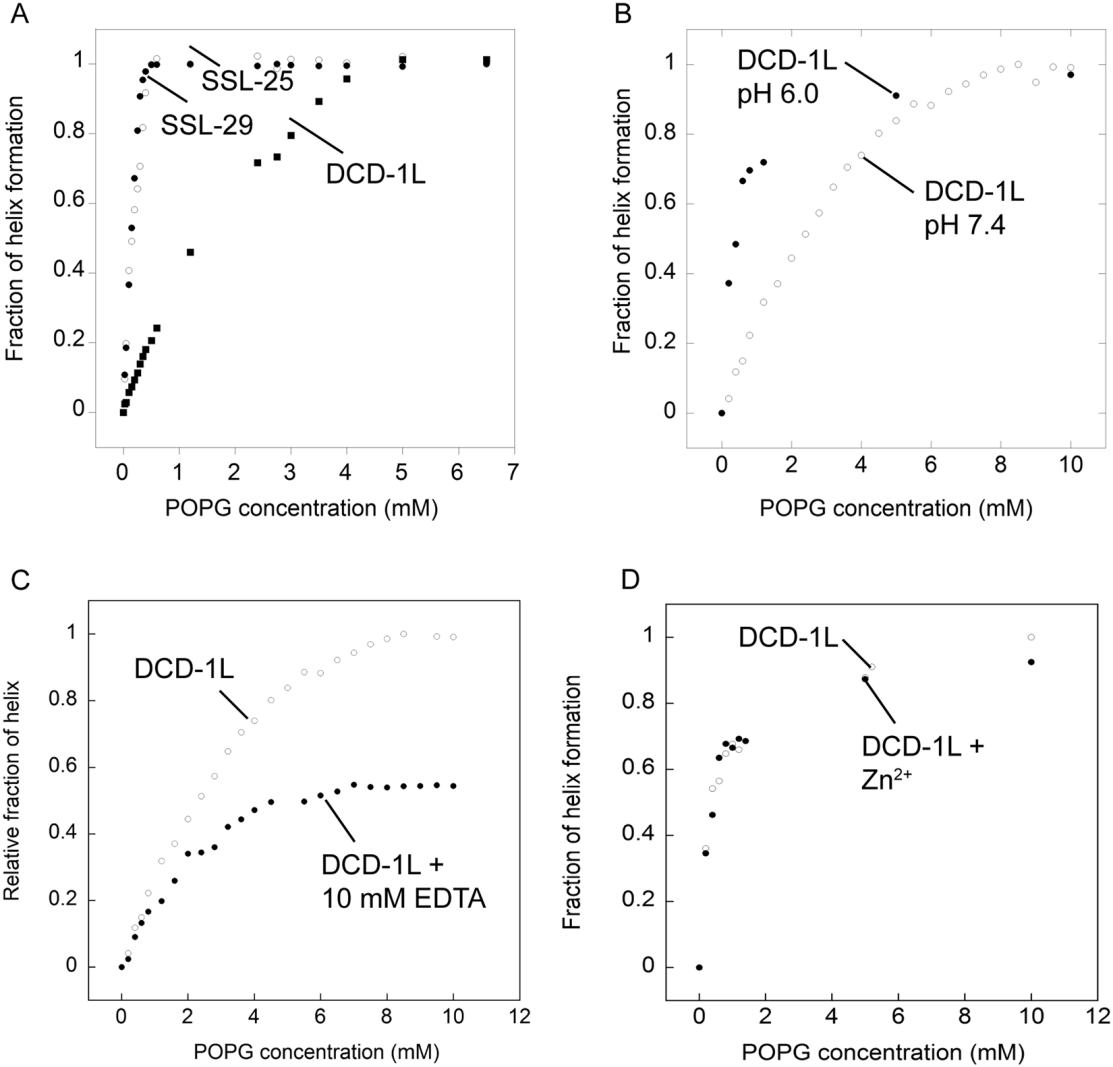
Affinities of DCD peptides to POPG monitored by changes in helical content using Far-UV CD signal at 218 nm. (**A**) Fractions of helical formation of SSL-25, SSL-29 and DCD-1L in the presence of different concentrations of POPG vesicles. “Fraction of helix formation” represents the ratio of helical content at a particular POPG concentration to the saturated helical content of the same molecule under the same buffer conditions. (**B**) Effect of pH on the affinity of DCD-1L to POPG vesicles. (**C**) Effect of EDTA on the affinity of DCD-1L to POPG vesicles at pH 7.4. “Relative fraction of helix” represents ratio of helical content at a particular POPG concentration to the saturated helical content of DCD-1L in POPG without EDTA. (**D**) Effects of the addition of Zn^2+^ on the affinity of DCD-1L to POPG vesicles at pH 7.4.

The DCD peptides are mostly unstructured in aqueous buffer and are only induced to form an α-helix when making contact (interacting/binding/inserting into) with detergent micelles or POPG vesicles. We next monitored the helical content (Far-UV CD signal at 218 nm, which produced biggest difference in signals between POPG bound and unbound forms) of DCD peptides by titrating DCD peptides with increasing concentrations of POPG vesicles: this allowed us to indirectly deduce the “affinity” of the DCD peptides with POPG. The titration results showed that both SSL-25 and SSL-29 reached close to 0.99 fraction of helix formation with 0.5 mM POPG (Fig. 3A), whereas DCD-1L required 5 mM POPG before reaching saturation. These results suggest that DCD-1L has a lower affinity for POPG than SSL-25 and SSL-29 in 20 mM Tris-HCl buffer at pH 7.4.

To determine if the binding of DCD-1L to POPG is pH dependent, we studied the binding of DCD-1L to POPG at pH 6.0, and found that the affinity is relatively higher compared with that at pH 7.4; albeit, the saturated α-helical contents are similar at both pH values (Fig. 3B). The affinity of DCD-1L to POPG at pH 6.0, however, is still lower than that of SSL-25 and SSL-29 at pH 7.4.

It was previously reported that Zn^2+^ or other divalent metal ions are essential for the membrane binding affinity of DCD-1L^15^. To determine if metal ions are essential for the affinity of DCD-1L to POPG, binding was studied in the presence of 10 mM EDTA. The results showed that the affinity of DCD-1L to POPG and the saturated α-helical content are significantly reduced in the presence of EDTA, suggesting that metal ions — inherited from the preparation of the recombinant protein and not necessarily Zn^2+^ — played a key role in DCD-1L binding to lipid (Fig. 3C). The addition of Zn^2+^ has a negligible effect in DCD-1L–POPG affinity, suggesting that the recombinant protein is already bound with metal ions or cannot bind Zn^2+^ ions (Fig. 3D). The C-terminal domain of DCD-1L alone (residues E30 to L48), however, is not sufficient to interact with POPG or be induced to form α-helical conformation in the presence of POPG (see Supplementary Fig. S2). The cationic N-terminal domain (SSL-25) is crucial and self-sufficient for binding to the negatively charged POPG vesicle.

### Effect of site-directed mutation of charged residues on POPG binding by DCD-1L

**Figure 4:**
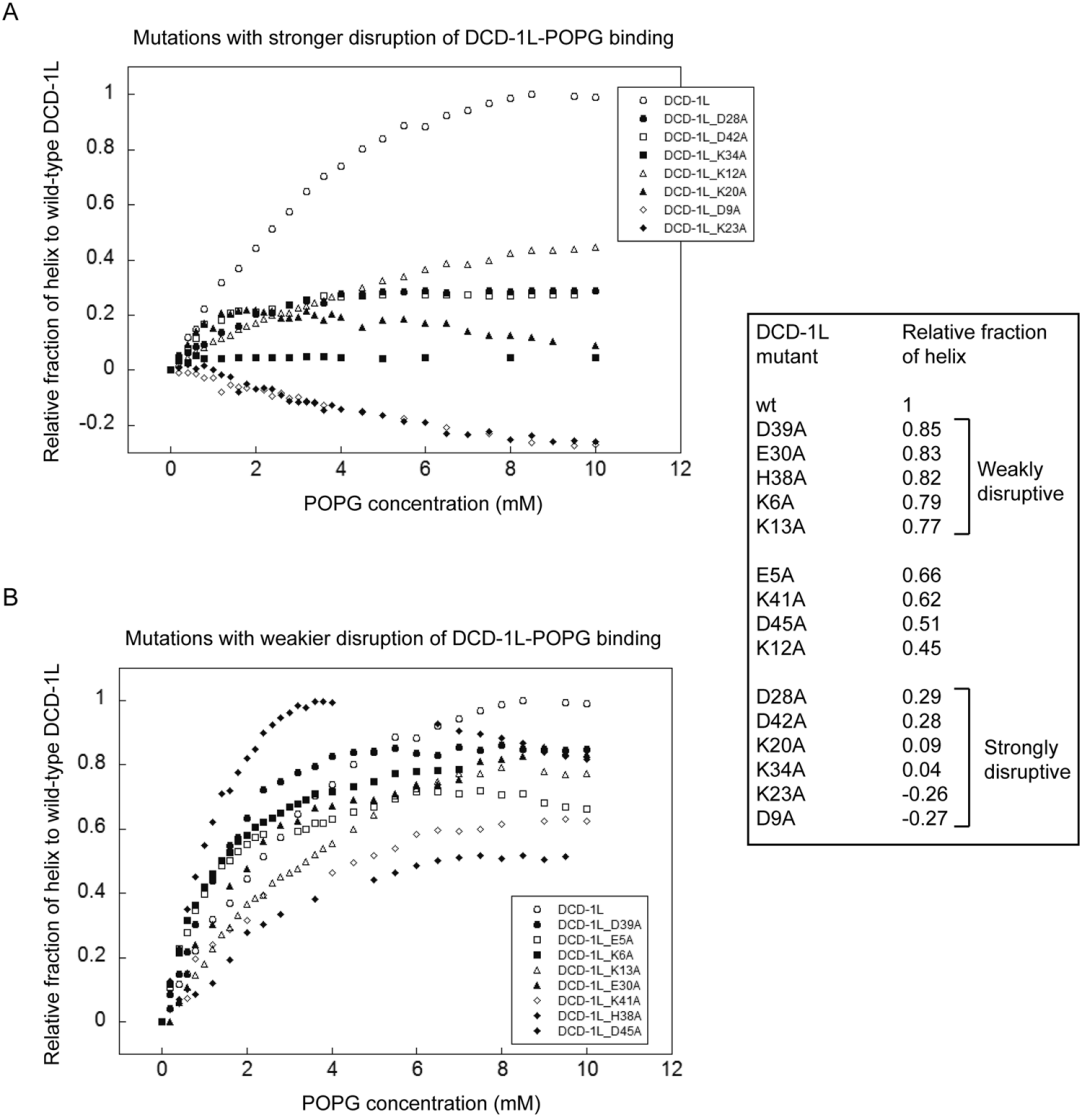
Affinities of site-directed mutants of DCD-1L to POPG as monitored by changes in helical content based on Far-UV CD signal at 218 nm. The relative fraction of helix of the site-directed mutants to that of the wild-type DCD-1L is plotted against the POPG concentrations. The site-directed mutants are classified into two groups; mutations that caused the relative fraction of helix at saturated POPG concentration to drop to below 0.3 are “strongly disruptive” mutations (**A**) and those above 0.7 are “weakly disruptive” (**B**). Other site-directed mutants have relative fraction of helix ranging from 0.44 to 0.66. Negative relative fraction of helix means that the CD signal at 218 nm is of the mutant is reducing in the presence of POPG.

The role of individual charged residue of DCD-1L in POPG binding was determined by comparing the relative fraction of helix formed by DCD-1L harboring point mutations versus the wild-type protein (Fig. 4). Each of the DCD-1L mutants investigated showed a reduction in the saturated helical content as compared with that of the wild-type. Some of the mutants (D9A and K23A) even showed a reduction in helical content in the presence of POPG, thereby producing a negative relative fraction of helix. We were able to classify the DCD-1L mutants into two groups: those that caused the relative fraction of helix to drop but above 0.5 (Fig. 4A) and those that caused the relative fraction of helix to drop below 0.5 or even a negative fraction of helix (Fig. 4B) as compared with the wild-type DCD-1L. To increase the stringency of this classification, mutations that caused the relative fraction of helix at saturated POPG concentration to drop below 0.3 were classified as “strongly disruptive”, whereas those above 0.7 were classified as “weakly disruptive” (Fig. 4 table insert). Based on this classification, mutations to charged residues, D9, K20, K23, D28, K34 or D42, were considered “strongly disruptive” to POPG binding. On the other hand, mutations to charged residues, K6, K13, E30, D39 or, interestingly, H38, were considered as only “weakly disruptive” to POPG binding (Fig. 4 table insert).

**Figure 5:**
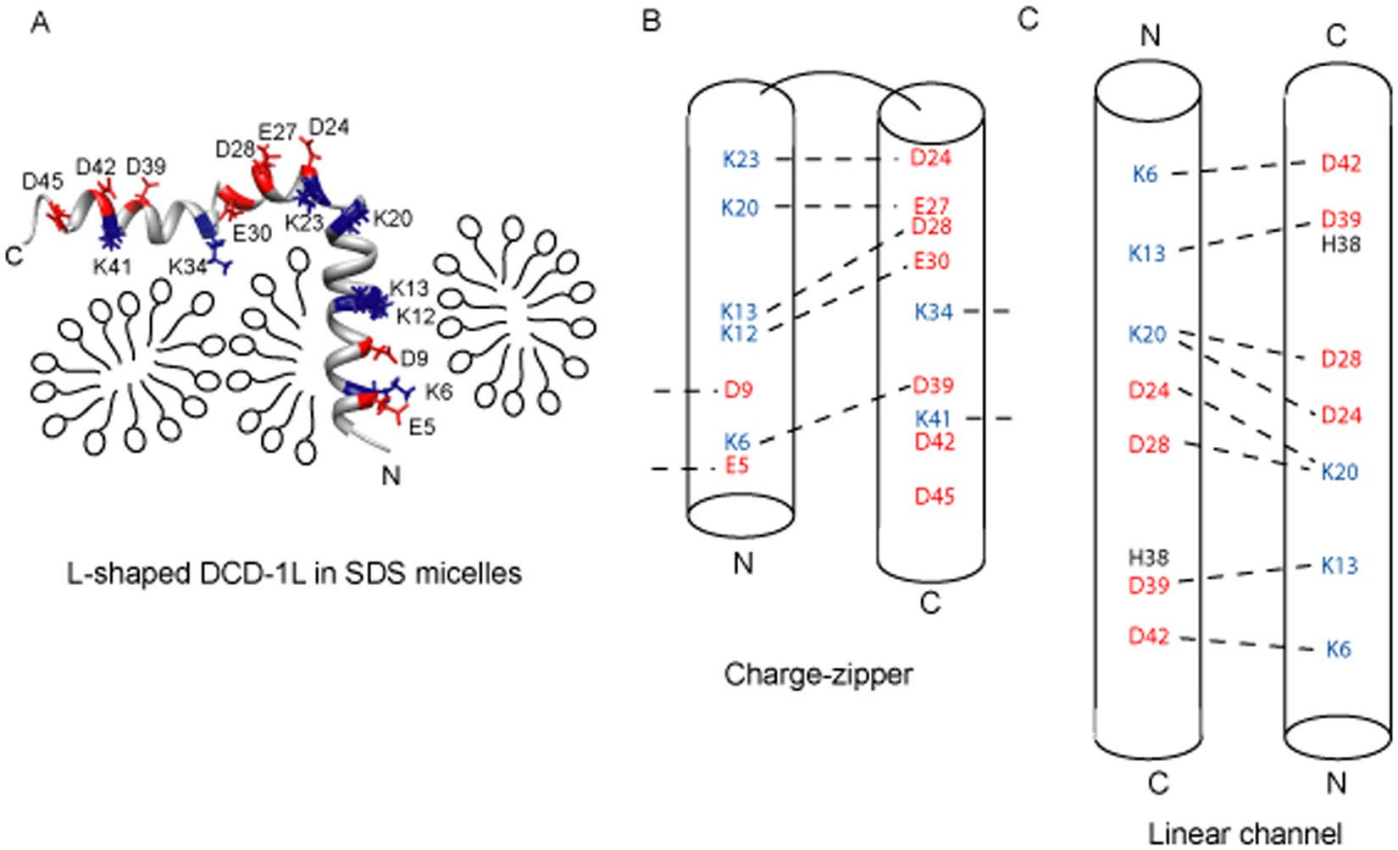
The three proposed models of DCD-1L when inserted into the lipid membrane. (**A**) The structure of DCD-1L in SDS micelles support a proposed model in which the positively charged N-terminal region of the “L-shaped” DCD-1L is inserted into lipid membrane, leaving the negatively charged C-terminal region floating on membrane surface; (**B**) The cationic N-terminal region of DCD-1L is folded back toward the anionic C-terminal region to form an “electrostatic charge-zipper”; and (**C**) DCD-1L is straight and tilted to keep the “linear channel” entirely within the membrane. The positively and negatively charged residues are colored blue and red, respectively. Dotted lines represent the proposed intra- and inter-molecular salt-bridges.

Our results support a model proposed in which the N-terminal domain of DCD-1L is inserted into the membrane, whereas the C-terminal domain of DCD-1L remains floating on the membrane surface^15^ (Fig. 5A). Positively charged residues at the C-terminal region, such as K20, K23 and K34, are essential for POPG binding and may interact with the negatively charged head groups of the membrane lipid. In contrast, positively charged residues at the N-terminal region, such as K6 and K13, are not essential for POPG binding and may be involved only in channel formation. This suggests that positively charged residues at the N- and C-terminal regions of DCD-1L may carry out distinct functions in POPG binding. Some of the negatively charged residues, such as D9, D28 and D42, when mutated, will strongly affect POPG binding. Other negatively charged residues, such as E30 and D39, are also exposed to the solvent but their mutations do not have a significant effect on lipid membrane binding.

In the proposed “charged-zipper” model, oppositely charged residues from the N- and C-terminal domains of DCD-1L form salt-bridges with each other within the same molecule or with adjacent molecules to stabilize the zipper for membrane insertion^16^ (Fig. 5B). Our site-directed mutagenesis analysis disagrees with this model, as residues that are involved in salt-bridge formation, e.g., K6, K13, E30 and D39, are only weakly disruptive to POPG binding when mutated. In contrast, mutations to residues involved in the formation of other salt-bridges, such as K20, K23 and D28, are strongly disruptive to POPG binding. Intriguingly, a salt-bridge is created by K13 and D28, however, it is unclear why one residue (i.e. D28) of the salt-bridge has a distinctively different effect when mutated as compared with the other residue (i.e. K13). In addition, we cannot explain why some acidic residues that are not supposed to be involved in salt-bridge formation, e.g. D42, are strongly disruptive to membrane binding when mutated (Fig. 5B). The results suggest that the “charged-zipper” model may not be correct, and that the salt-bridges are not the essential forces for membrane insertion by DCD-1L.

In the proposed “linear channel” model (based on the crystal structure of DCD-1L), intermolecular salt-bridges at the dimeric interface of the trimer are involved in stabilizing the channel. The residue H38 is also proposed to coordinate Zn^2+^ and be essential for membrane binding^13^ (Fig. 5C). However, our site-directed mutagenesis results also disagree with this model. Charged residues D9, K23 and K34 are not supposed to be involved in salt-bridge formation in this model, but their mutations caused significant disruptions to POPG binding by DCD-1L. In contrast, charged residues K6, K13 and D39 are supposed to form important salt-bridges in this model, but their mutation only weakly affected POPG binding. Furthermore, residue H38 can also be mutated without significantly affecting POPG-induced helix formation, suggesting that it may not be the critical residue for POPG binding.

### Effect of Zn^2+^ in the aggregation of DCD-1L

The oligomerization of DCD peptides are proposed to be essential for their antimicrobial activities and membrane pore formation, and metal ions, especially Zn^2+^, are required for this oligomerization step^15^. NMR titrations were used to investigate the role of Zn^2+^ in the aggregation of DCD peptides in both the unfolded free form and the helical SDS-bound form at different pH values: metal binding to proteins is affected by pH. NMR titrations were performed sequentially, by first recording the 1D NMR spectra of DCD peptides in the absence and then in the presence of Zn^2+^ to observe the effect of Zn^2+^ on aggregation of the free form of the DCD peptides, followed by the addition of SDS to induce the helical conformation, and then EDTA to remove Zn^2+^ to study the role of Zn^2+^ in aggregation of the SDS-bound form of DCD peptides.

**Figure 6:**
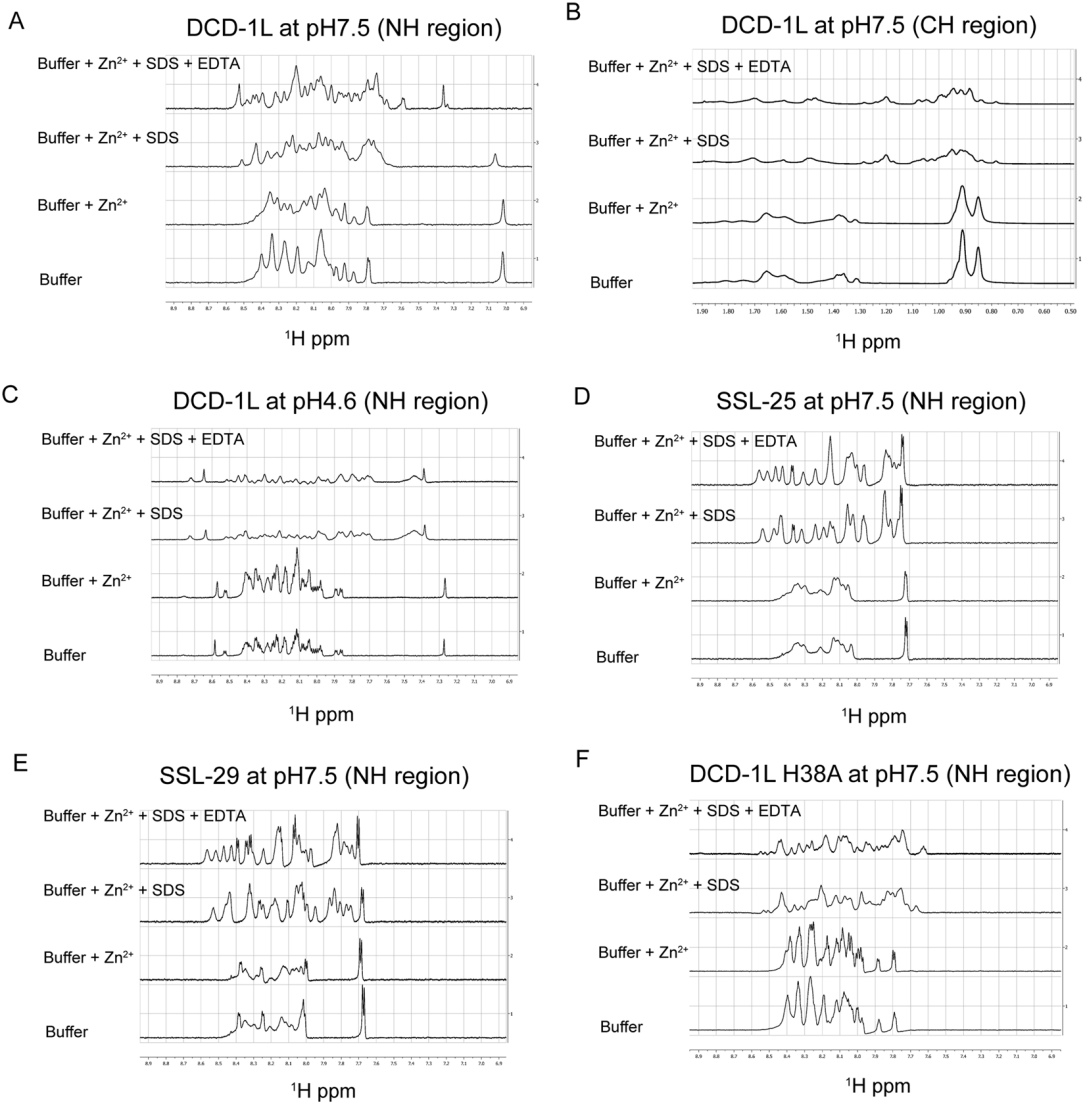
Effects of Zn^2+^ and SDS on the aggregation and conformational changes of DCD-1L as monitored by titration using 1D NMR spectra. Each diagram contains four 1D NMR spectra acquired using the same 500 μM peptide sample (500 μl) in the following orders: “Buffer”: either 20 mM sodium acetate at pH 4.6 or 20 mM Tris-HCl at pH 7.5; “Buffer + Zn^2+^”: 0.5 μl of 0.5 M ZnCl_2_ was added to a final concentration of 0.5 mM Zn^2+^; “Buffer + Zn^2+^  + SDS”: 25 μl of 10% deuterated SDS was added to a final concentration of 0.05% SDS; and “Buffer + Zn^2+^  + SDS + EDTA”: 1 μl of 0.5 M EDTA was added to a final concentration of 1 mM. The samples used were: (**A**) and (**B**) DCD-1L at pH 7.5, showing the NH and CH regions, respectively, of the 1D NMR spectra; (**C**) DCD-1L at pH 4.6, showing the NH region of the 1D NMR spectra; (**D**) SSL-25 at pH 7.5, showing the NH region of the 1D NMR spectra; (**E**) SSL-29 at pH 7.5, showing the NH region of the 1D NMR spectra; and (**F**) DCD-1L His38Ala mutant at pH 7.5, showing the NH region of the 1D NMR spectra.

We found that, in the absence of SDS and Zn^2+^, DCD-1L is unfolded with sharp and non-dispersed peaks in the NH region of the 1D NMR spectrum (Fig. 6A). In the presence of Zn^2+^, the peaks become broader and more dispersed, suggesting aggregation and partial folding. The peaks are further dispersed as DCD-1L changes to a helical conformation upon the addition of SDS. The subsequent removal of Zn^2+^ causes an increase in the linewidth of the peaks, suggesting the formation of the monomeric and helical species (Fig. 6A). A similar pattern of changes in the linewidth and separation of the peaks were also observed in the CH region of the 1D NMR spectrum, which is less prone to artefacts (such as amide proton exchange, pH and temperature variations) than the NH region (Fig. 6B).

To investigate if pH also plays a role in metal binding and thus the aggregation and activity of DCD-1L, the NMR titration experiment was performed at an acidic pH 4.6. The results showed that the Zn^2+^ binding was significantly affected by an acidic pH, with no DCD-1L aggregation observed upon the addition of Zn^2+^ (Fig. 6C). Although DCD-1L can still form a helical and folded conformation in the presence of SDS at this acidic pH, this is only in a monomeric form, and the removal of Zn^2+^ by EDTA had no effect on the linewidth or separation of the peaks (Fig. 6C).

To determine if the C-terminal domain (acidic) is essential for Zn^2+^ binding and aggregation of DCD, the same sequential NMR titration experiments were performed using SSL-25 (Fig. 6D), SSL-29 (Fig. 6E) and DCD-1L harboring a H38A substitution (Fig. 6F). We found that both SSL-25 and SS-29 aggregate, even in the absence of Zn^2+^. Unlike DCD-1L, the addition of Zn^2+^ to SSL-25 and SSL-29 did not further increase the aggregation. The NMR spectrum of the DCD-1L H38A mutant behaved differently to that of wild-type DCD-1L, but was similar to SSL-25 and SSL-29, with the addition of Zn^2+^ having no enhancing effect on the aggregation. SSL-25, SSL-29 and DCD-1L H38A all converted to a helical conformation in the presence of SDS: however, the later removal of Zn^2+^, had no effect on the aggregation state of these peptides.

Overall, these results suggest that the C-terminal domain of DCD-1L, which contains most of the acidic charged residues and H38, is essential for Zn^2+^ binding and aggregation of DCD-1L, in both the free and SDS-bound forms.

## Discussion

Antimicrobial peptides are an integral part of the innate defense system of the skin and may play a role in regulating the normal skin flora. Previous studies have sought to understand the antimicrobial actions of DCD-1L through structural analyses, but have yet to provide a definitive mode of action for this protein. Here, we used NMR, Far-UV CD, and mutational analyses to clarify the insertion of DCD-1L into bacterial membranes. We show that DCD-1L interacts with SDS and POPG, but not POPC, vesicles, suggesting that membrane binding is a specific interaction that requires the presence of a negatively charged lipid head group. Furthermore, the helical conformation in SDS and POPG differs significantly from that in 50% TFE^12^, suggesting that DCD-1L, when inserted into the bacterial membrane, adopts a conformation with a higher helical content than previously believed.

We observed distinct biochemical properties for the N- and C-terminal regions of DCD-1L. The N-terminal region is amphipathic, with both positively and negatively charged residues on one side and the other side is entirely hydrophobic. This region is proposed to be inserted into the membrane and lining the ion channel^14^. We found that the N-terminal region alone (SSL-25, SSL-29) is sufficient to interact with POPG vesicles. SSL-25 and SSL-29, which lack the C-terminal region, show higher binding affinities to POPG, suggesting that the C-terminus of DCD-1L likely acts as a regulatory domain to inhibit the binding of DCD-1L to lipid membranes. The C-terminal region of DCD-1L (from residues D24 to D45) is mostly composed of acidic residues (7 out of 9 charged residues) with only two positively charged residues. When the pH is high, it is plausible that the C-terminal region could repel the negatively charged bacterial membrane. Indeed, in lower pH conditions, binding of DCD-1L to POPG vesicles is much stronger. Others report similar pH-dependent changes^14^. Using a mercury-supported lipid membrane with self-assembling DOPS monolayer, Becucci and others report that DCD-1L is neutral and can permeabilize the membrane at the physiological transmembrane potential at pH 5.4 but, at pH 7.0, it is negatively charged and can only permeabilize the membrane outside the physiological transmembrane potential^14^.

Zn^2+^ and metal ion binding is necessary for the proper insertion of DCD-1L into lipid membrane. The binding affinity between DCD-1L and SDS was significantly lower in the presence of EDTA as compared to the control. Becucci *et al*. found that DCD-1L is negatively charged and con only permeabilize the membrane outside the physiological transmembrane potential at pH 7.0. In the presence of Zn^2+^, however, DCD-1L can permeabilize the membrane, even at pH 7.0 and physiological potential^14^. Zn^2+^ is proposed to induce self-assembly of DCD-1L (peptide-peptide or peptide-lipid salt bridges) upon interaction with lipid bilayer, which is a pre-requisite for ion-channel formation^15^. Although the C-terminal region forms a helical structure when DCD-1L binds POPG or SDS, the C-terminus alone cannot be induced to form helical structure in POPG or SDS. This suggests that the N-terminal region initiates the membrane insertion and helix formation, which in turn leads to the subsequent helical conformation at the C-terminal region. Indeed, in SSL-25 and SSL-29, which lack a C-terminal region, the N-terminal region is self-sufficient for helix formation in the presence of SDS without Zn^2+^. When the C-terminal region is entirely removed (i.e., SSL-25 and SSL-29), Zn^2+^ does not induce aggregation of the peptides, in either the free or SDS-bound states. Based on the line widths of the NMR spectra, we suspect that SSL-25 and SSL-29 aggregate in solution by themselves independent of Zn^2+^ and perhaps through a novel mechanism. SSL-25 and SSL-29 represent a different spectrum of antimicrobial activity as compared with DCD-1L, perhaps acting at different regions of the body.

**Figure 7:**
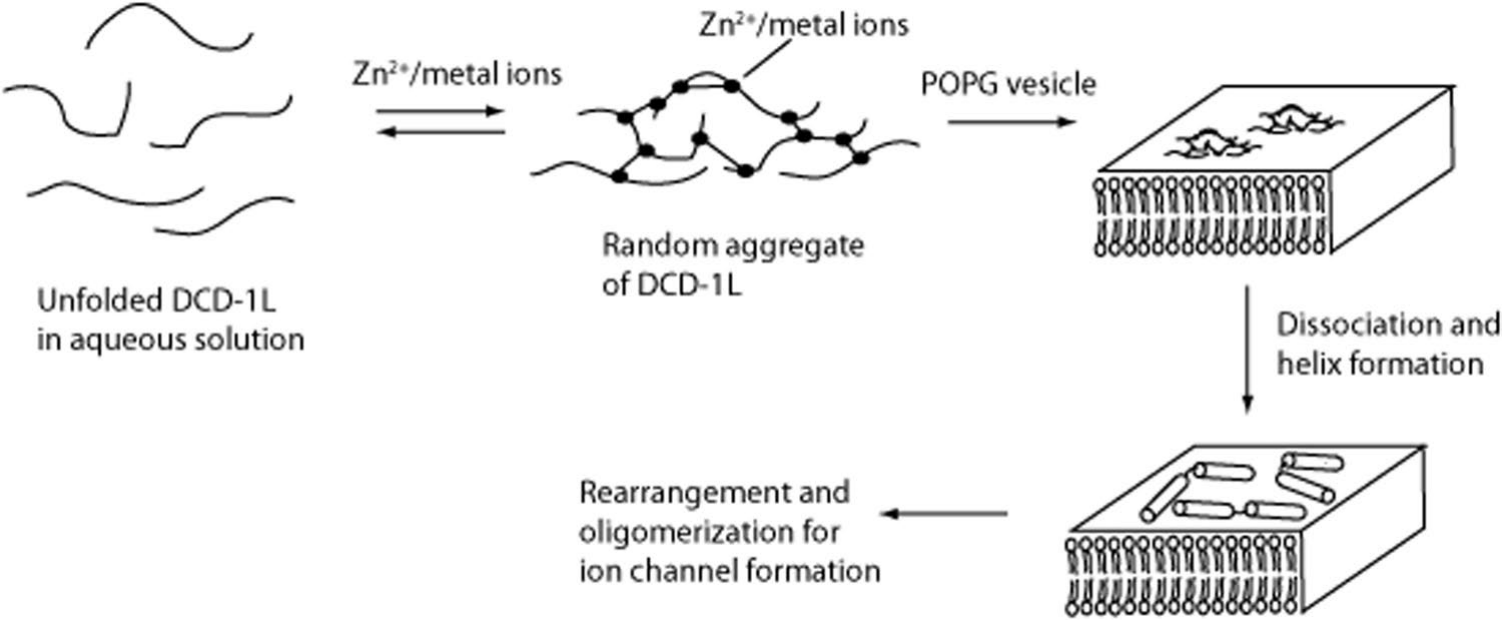
Supported model of lipid membrane insertion by DCD-1L. Curved lines represent unfolded DCD-1L in solution. Closed circles represent metal ions (or Zn^2+^); cylinders represent the N-terminal and C-terminal helical regions of DCD-1L in the presence of a lipid membrane (open circles with two tails); and straight lines represent electrostatic bonds formed between metal ions and DCD-1L.

Our 3D structure shows that DCD-1L in the presence of 30 mM SDS assumes an “L-shaped” conformation, with three helices forming two regions. Based on our results, we support a model proposed for DCD-1L membrane insertion^15^ as shown in Fig. 7. DCD-1L is largely unfolded in aqueous solution. DCD-1L forms random aggregates upon binding with Zn^2+^ or metal ions. Our results agree with a model in which aggregated DCD-1L clusters are absorbed on the surface of a lipid bilayer. The interactions are mediated through negatively charged residues at the C-terminus, and, as indicated above, are likely affected by the environmental pH. These random aggregates with less negative charge bind to lipid bilayer, and are induced to form a helical conformation. Formation of helices will likely cause dissociation of DCD-1L from the aggregate. The monomers then rearranged and oligomerized to form ion channels stabilized by metal cations^15^. The aggregation by Zn^2+^ (or metal ions) enhances and induces lipid membrane binding, and requires the H38 residue in the C-terminal region; when H38 is mutated to Ala, Zn^2+^ can no longer induce aggregation of DCD-1L both in the free or SDS-bound helical form.

Two other models have been proposed in earlier studies. The first model depicts a tilted membrane channel was proposed as a way to account for the straight hexameric α-helical bundle determined in the crystal structure of DCD-1L in the absence of a membrane or lipid (“linear channel”)^13^. The second (“charged zipper”), describes a trans-membrane hairpin model has also been proposed based on the pattern of salt-bridges in the “electrostatic charge-zipper” model^16^. However, we did not observe such straight helices or any hairpin structures for DCD-1L in the presence of SDS. Furthermore, in the proposed “charge-zipper” or “linear channel” models, salt-bridges are employed to stabilize the overall structure of the assembly (Fig. 5). However, our mutational analysis showed that substituting these charged residues (either positively or negatively charged) rendered different effects depending on the residue, implicating the likelihood of other functions for these charged residues. As outlined above, we show that the charged residues at each region carry out distinct functional roles, with minimal involvement in the formation of intra-molecular and inter-molecular salt bridges. Indeed, we show that some of residues not previously implicated in salt-bridges, e.g. K44 and D45, were seemingly essential for lipid binding and may bind Zn^2+^ for stabilization of the ion channel.

DCD-1L is a vital component of the epithelial innate immunity. Understanding the structure and mechanism of the membrane insertion of DCD peptides could lead to development of a new class of non-cationic antibacterial peptides. Our mutational study support a model proposed for membrane insertion that involves partial membrane insertion, such that the N-terminal region of DCD-1L forms a toroidal pore, while the C-terminal region remains floating on surface^15^. In this model, the N- and C-terminal regions carry out different functional roles, with minimal salt-bridges formed. The N-terminal charged residues may be involved in lining the ion channel. The negatively charged residues at the C-terminus may help to stabilize the channel and coordinate the interactions with metal ions, whereas the positively charged residues interact with the negative head groups of the lipids.

## Materials and Methods

### Cloning of DCD gene

The codon optimized DNA insert was amplified by PCR using a forward primer containing a *Bam*H I restriction site and a factor Xa digestion site, and a reverse primer containing an *Eco*R I restriction site. PCR fragments were amplified using KOD Hot Start polymerase (Novagen; Madison, WI) and the product quality was confirmed on a 1% agarose gel. The DNA band of correct size was excised and purified using a Gel Extraction Kit (QIAGEN; Valencia, CA) according to the standard manufacturer’s protocol. The purified DNA insert (~100 ng/μl) was digested using 1 μl (10 U) of *Bam*HI FastDigest^TM^, 1.5 μl (10 U) and *Eco*RI FastDigest^TM^ (Fermentas; Vilnius, Lithuania) 37 °C for 2 h. The plasmid vector (pGEX-4T-1) was prepared separately using the same protocol. The digested DNA insert and plasmid vector were mixed and purified using QIAGEN spin columns and subsequently ligated using T4 DNA ligase at room temperature for 2 h. The ligated plasmids were then transformed into DH5α competent cells.

### Site-directed mutagenesis

Site-directed mutagenesis was performed using PCR. For each single mutant, we designed a pair of complementary primers with mismatched bases in the middle. PCR was carried out using high-fidelity KOD Hot Start DNA Polymerase (Novagen; Madison, WI) synthesizing the whole plasmid, including the mismatched bases. The methylated wild-type plasmids were digested using 1 μl of 1 U *Dpn*1 FastDigest^TM^ at 37 °C for 1 h. The mutated plasmids were subsequently transformed into DH5α competent cells and plated onto LB agar with ampicillin at 37 °C for at least 16 h. The mutated plasmids were then verified by DNA sequencing.

### Protein expression and purification

The expression vector containing the desired DNA insert was transformed into BL21 (DE3) cells. A single colony was inoculated into LB medium with 100 μg/ml of ampicillin and incubated overnight at 37 °C. The overnight culture was transferred into 1 L LB containing 100 μg/ml of ampicillin and grown at 37 °C until the OD_600_ reached 0.6. Recombinant protein expression was induced with 0.35 mM isopropyl β-D-1-thiogalactopyranoside (IPTG) for 16 h at 20 °C. The cells were harvested and resuspended in 1 × PBS buffer (140 mM NaCl, 2.7 mM KCl, 10 mM NH_2_PO_4_ and 1.8 mM KH_2_PO_4_, pH 7.4). For the ^15^N- and^13^C-labeled protein samples, 1 g/L of ^15^N ammonium chloride and ^13^C glucose was used in M9 minimal media following similar expression conditions. The recombinant protein was GST-tagged and purified using glutathione-sepharose affinity chromatography. The GST-tag was cleaved at room temperature using Factor Xa protease (0.5 U/mg protein) at a designed site that leaves no extra residue at the N-terminus of the peptide. The digested mixture was further purified using HiLoad 16/60 Superdex 75 pg column (GE Healthcare; Buckinghamshire, UK).

### Circular dichroism

FarUV-CD experiments were conducted with 30 µM of protein in 20 mM Tris-HCl, pH 7.5 and 0.1 M NaCl at room temperature. FarUV-CD spectrums were acquired using Jasco J-810 spectropolarimeter (Jasco, Japan) with a Hellma quartz cuvette of 1.0 mm path length. The spectrums were recorded at a wavelength range of 190–260 nm with 0.1 nm resolution using a scan speed of 50 nm/min and averaged for 10 scans. Lipid binding was carried out using a reference wavelength at 218 nm. POPG and POPC (Avanti; Alabaster, AL) were dissolved in chloroform at a concentration of 25 mg/ml (stored at −20 °C before use), and 0.5 ml was transferred to a glass tube. The chloroform solvent was removed by blowing a slow stream of nitrogen gas over the chloroform solution. The obtained lipid film was further dried under vacuum overnight to remove trace solvent. A suitable buffer was added to the glass tube and incubated at 4 °C overnight. The solution was then mixed thoroughly and sonicated at 4 °C until the solution appeared transparent. The size and the homogeneity of the vesicles were measured using dynamic light scattering. The vesicle size obtained was around 60 to 100 nm. The concentration of lipid was calculated based on the amount of lipid used for preparation, the molecular weight of the lipids, and the volume of the buffer.

### NMR experiments and structure calculation

Recombinant ^15^N- and/or ^13^C-labeled DCD-1L were prepared in 60 mM SDS without Zn^2+^ for NMR structure determination. 2D ^1^H-^15^N HSQC spectra were acquired to obtain ^1^H-^15^N correlation peaks. 3D heteronuclear NMR experiments, including HNCACB^17^, CACB(CO)NH^18^, CC(CO)NH, HCC(CO)NH^19^ and HCCH-TOCSY^20^, were used to assign the backbone and side-chain chemical shifts. NOE restraints were obtained from the 3D ^15^N-NOESY^21^ and^13^C-NOESY^22^. All NMR experiments were performed using a Bruker AVANCE 800 MHz spectrometer equipped with a cryoprobe. The obtained NMR data were processed using NMRPipe and NMRDraw^23^. The TALOS program was used to predict the angular restraints for the structural calculation of DCD-1L^24^. The processed NMR data were then analyzed using SPARKY software^25^. The backbone and side-chain resonance assignments of DCD-1L were deposited at the Biological Magnetic Resonance Bank. The 3D structures of DCD-1L were calculated using the CNS software^26^. The final 20 structures were checked with Procheck-NMR^27^ and deposited at the Protein Data Bank. For titration using 1D NMR, four different peptides DCD-1L, DCD-1L_H38A, SSL-25 and SSL-29 were commercially synthesized (Genscript), and diluted into two different buffer solutions (20 mM sodium acetate pH 4.6 or 20 mM Tris-HCl pH 7.5) to a final concentration of 500 μM. The 1D NMR spectrum (32 scans) was first measured for each peptide in its specific buffer. After the first experiment, 0.5 μl of ZnCl_2_ (0.5 M) was mixed with the sample, and another 1D spectrum was acquired, followed by the addition of 25 μl of 10% deuterated SDS and the acquisition of another 1D NMR spectrum. Finally, 1 μl of EDTA (0.5 M) was added, and the final 1D spectrum was acquired. These steps were repeated for all the four peptides.

### PDB and BMRB deposition

The NMR structure of DCD-1L is deposited at PDB with ID of 2ndk. The backbone and side-chain resonance assignments of DCD-1L are deposited at the Biological Magnetic Resonance Bank with accession number: 26063.

## Electronic supplementary material

Supplementary materials
