# Supplementary material for: Structural basis for the bacterial membrane insertion of dermcidin peptide, DCD-1L
Source: Sci Rep. 2017 Oct 24;7:13923. doi: 10.1038/s41598-017-13600-z (PMC5654962; doi:10.1038/s41598-017-13600-z)
Supplement: Supplementary file 1 — Supplementary materials [file 41598_2017_13600_MOESM1_ESM.doc]

**Structural basis for the bacterial membrane insertion of dermcidin peptide, DCD-1L**

**Van Sang Nguyen, Kang Wei Tan, Karthik Ramesh, Fook Tim Chew and Yu Keung Mok**

Department of Biological Sciences, 14 Science Drive 4, National University of Singapore, Singapore 117543

Supplementary table and figures

| 1. **Restraints used for calculation** |  |
| --- | --- |
| Total NOEs | 607 |
| Intraresidues (i,j) | 214 |
| Interresidue, sequential (|i, j| = 1) | 246 |
| Interresidue, medium range (1< (|i-j| ≤ 5 ) | 147 |
| Interresidue, long-range (|i-j| ≥ 5 | 0 |
| Dihedral angle restraints (φ, ψ) | 65 |
| Hydrogen bond restraints | 0 |
| **B. Structure statistics** |  |
| No. of distance violations of > 0.20 Å | 2 |
| No. of dihedral angle violations of > 4° | 0 |
| Max. dihedral angle violation (°) | 3.5 |
| Max. distance constraint violation (Å) | 0.36 ± 0.007 |
| Average RMS deviation (Å): |  |
| Back bones (Full length) | 2.36 +/- 0.88 |
| Heavy atom (Full length) | 2.80 +/- 0.85 |
| Back bone atoms (residues 2-18) | 0.52 +/- 0.24 |
| Back bone atoms (residues 20-30) | 0.23 +/- 0.11 |
| Back bone atoms (residues 32-47) | 0.69 +/- 0.27 |
|  |  |

**Supplementary Table S1.** Structural statistics of the NMR structure of DCD-1L


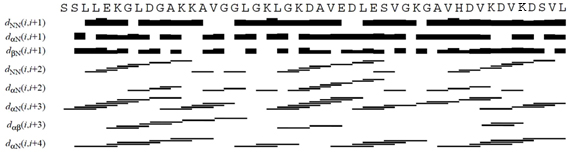


Supplementary Figure S1. Relative NOE intensities and connectivities from ^15^N and ^13^C-edited NOESY of DCD-1L


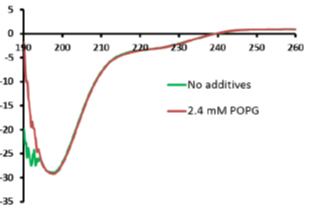


Supplementary Figure S2. Far-UV CD spectra of C-terminal region of DCD-1L from residues Glu30 to Leu48 in the absence (green) and presence of 2.4 mM POPG (red) in 10 mM phosphate buffer at pH 7.4.
